# The requirements of providing health education for rural people through electronic methods: the experiences and perspectives of community health workers

**DOI:** 10.1186/s12909-024-06073-y

**Published:** 2024-09-30

**Authors:** Seyed Hadi Hosseini, Zahra Tayebi, Mohammad Amerzadeh, Behrooz Pouragha, Effat Hatefnia, Shahab Moeini-Mostofi, Seyedeh Sedreh Hosseini, Yasaman Poormoosa

**Affiliations:** 1 Department of Healthcare Services Management, School of Health, Alborz University of Medical Sciences, Karaj, Iran; 2 Department of Nursing, School of Nursing, Alborz University of Medical Sciences, Karaj, Iran; 3 Non-communicable Diseases Research Center, Research Institute for Prevention of Non-Communicable Diseases, Qazvin University of Medical Sciences, Qazvin, Iran; 4 Department of Health Promotion and Education, Social Determinants of Health Research Center, School of Health, Alborz University of Medical Sciences, Karaj, Iran; 5 Deputy of development, management and resources, Alborz University of Medical Sciences, Karaj, Iran; 6 Department of Exceptional Student Education, General Department of Education of Alborz Province, Karaj, Iran

**Keywords:** Health education, E-learning, Community health workers, Behvarz

## Abstract

**Background:**

Reliance solely on traditional approaches in health education is no longer considered sufficient, and electronic/digital education can be a complementary approach. Implementing electronic methods in health education requires identifying the requirements from the perspective of the service providers. Therefore, this study aimed to elucidate the experiences and perspectives of community health workers (Behvarzan) regarding the requirements for providing health education for rural people through electronic/digital means.

**Methods:**

This descriptive qualitative study was conducted in 2022 at Alborz University of Medical Sciences. Data were collected through semi-structured interviews with 14 Behvarzan from Health Houses (HH) in Karaj City. The data were analyzed using the inductive Elo & Kyngas approach and conventional content analysis facilitated with MAXQDA software.

**Results:**

After analyzing the results, we extracted 139 open codes, and by merging them for more precise coding and to facilitate the research process, we formed three main themes and ten subthemes. The themes included Technology (technical infrastructure, content production, content delivery methods, and content delivery channels), Facilities and equipment (communication equipment and electronic content repositories), and Stakeholders (training of providers, motivating providers, persuading learners and target groups, and reference groups).

**Conclusion:**

From the perspective of rural healthcare workers, the implementation of electronic education requires necessary technology, equipment, facilities, processes, and content should be pursued and provided through specialized working groups, extending from the Ministry of Health and Medical Education to local HHs. These resources should be available to the healthcare workers and their target populations. Concurrently, educational programs and incentives should be defined and offered at the university level and within health networks for rural healthcare workers and their populations.

##  Background

One main group of service providers in the most peripheral layer of the health system structure is the community health workers (Behvarzan), directly responsible for providing primary health services (PHC) to the people in rural areas [1]. The Iranian Behvarz is a full-time employee of the Iranian health system selected from and resides within their local community. They operate in rural Health Houses (HHs), among the most important healthcare centers in Iran’s rural areas. Behvarz functions as the primary custodians of these rural health houses and are responsible for the population’s health in their designated area [2].

For every 1,500 rural inhabitants, one HH is established, which, depending on geographical conditions, covers one or more villages (satellite villages). Typically, each HH employs one female and one male Behvarz, who are responsible for providing primary healthcare services to approximately 2,000 individuals [3].

The target groups for Behvarzes in HHs are as follows:

1. Children: From birth to 4 years of age (height, weight, vaccination).
2. Adolescents: 5 to 17 years of age.
3. Young adults: 18 to 29 years of age.
4. Middle-aged adults: 30 to 59 years of age.
5. Elderly: 60 years and above (subdivided into 60–70 years and over 70 years).
6. Women and mothers: (pre-pregnancy, during pregnancy, postpartum)
7. Students (in schools).
8. Health ambassadors (volunteers from families).
9. Village environment (environmental health).
10. Active workshops, workers, and employees at the village level (occupational health) [4].

Behvarzan play a role in preserving and promoting their target groups’ health through implementing health education programs, considered one fundamental and successful strategy for health promotion [5, 6]. Appropriate performance is the fastest way to improve community health [7, 8]. Besides, providing health education based on people’s real needs increases the participation of local and public forces in rural areas and the acceptability and success of health services [2]. Behvarzan’s job description includes identifying the primary health issues and problems of the village, determining health priorities, planning educational activities in line with health priorities, preparing a scheduled educational timetable (Gantt chart), familiarizing with different teaching methods and implementing educational programs considering the target group, forming formal and informal groups for education, mobilizing public participation in health activities, forming health councils with key individuals in the village, following up on the decisions made in the health council meetings, providing education and properly distributing educational materials to the people in the region, and using appropriate educational media in implementing education. These tasks seem to have been developed with a traditional mindset. Therefore, the relevant training is mainly conducted by conventional methods such as face-to-face instruction and lecturing and with tools like brochures, pamphlets, and posters. However, in this approach, educational needs are not given much attention, and learner assessment is generally not conducted [9]. Nowadays, conventional education is insufficient to meet educational needs and manage complex affairs. Therefore, new and appropriate patterns and methods must be adopted to engage and enhance the effectiveness of education for the target audience and encourage service providers to play a more active and creative role [10]. Therefore, digital technologies have been introduced as an integral part of the work and educational environment and have generally changed the methods of teaching and learning so that the science and art of teaching-learning have also been integrated with the growth of information and communication technologies and a new approach to learning called e-learning has emerged in line with the society needs [11].

This educational method has created a learning environment based on the learner’s needs and has incorporated more flexibility into the educational process. E-learning, using the latest achievements of the information and communication technology era, has created new approaches and provided bright horizons in education for various fields [12].

E-learning refers to educational activities using electronic tools such as audio, visual, computer-based, and specifically internet-based [13, 14]. E-learning has introduced considerable flexibility in educational methodologies, content management, synchronous and asynchronous interaction between teachers and learners, course organization and structure, education plans, and learner evaluation [15]. It has also provided an opportunity for educating individuals who cannot receive in-person training due to lack of access to in-person educational centers, insufficient time, and spatial and temporal limitations [16].

Today, we are witnessing the rapid expansion of access to appropriate hardware and software for e-learning, such as the development of internet infrastructure and access to personal computers and smartphones in rural areas. Tamba et al. [17]. in Guinea and Thermizi et al. [18] in Afghanistan found the healthcare workers’ satisfaction with learning health-related issues electronically and in a blended manner, and they have found the use of mobile-based education to be influential in healthcare providers’ knowledge regarding specific diseases. Bertman et al. have also shown the high effectiveness of e-learning in controlling AIDS in Zimbabwe [19]. Using inexpensive Android tablets has also been very effective in Uganda for identifying, treating, and preventing pneumonia in children under five years. It has been highly satisfactory from healthcare workers’ perspective. Researchers have considered the blended learning approach a practical substitute for traditional learning in remote areas due to the limited human resources [20].

During the COVID-19 pandemic, an opportunity emerged as many individuals avoided visiting HHs and healthcare centers due to the rapid spread of the disease [21]. Consequently, Behvarzes were compelled to utilize electronic learning methods (in an unplanned manner) to educate the public on health-related matters [22]. Therefore, the research team sought to leverage this invaluable experience from a specific group (primary healthcare workers) to determine the requirements from their perspective if we systematically continue this educational approach. Naturally, having relevant experiences would be highly beneficial in identifying individuals’ viewpoints.

## Methods

### Study design

This study was conducted using a qualitative content analysis method in 2022 at Alborz University of Medical Sciences.

### Participants and recruitment

Purposive sampling was performed among the Behvarzan in HHs in the central district (Garmdare, Mohammad Abad, and Kamalabad villages) and Asara district (Adaran, Asara, and Nesa villages) of Karaj City. Villages in the central district are located on the southern slope of the Alborz mountain range. They are near the city, making physical access to the respective HHs easier. The villages in the Asara district are located in the middle of the Alborz mountain range, further away from the urban areas, and have more difficult physical access to the HHs. Interviewing the Behvarzan from districts with different geographical locations provided the opportunity to extract diverse perspectives influenced by their varied experiences.

The inclusion criteria were having at least two years of work experience in the HH, having at least one satellite village, using at least one e-health education method (based on the interviewer’s explanation) in providing health education, and willingness to participate in the study. The interviews with the Behvarzan were conducted from September to November 2022 until data saturation was reached. Ultimately, 14 Behvarzan participated in the study.

### Data collection

Before conducting the interviews, we extracted the guiding questions after reviewing the relevant studies and obtaining five expert faculty members’ opinions. We used the prepared interview guide in two pilot interviews and reviewed the results by several experts. The interview guide was then improved based on the research objective and used as the data collection instrument.

After matching Behvarzan’s conditions with the inclusion criteria, we explained the study objectives and set the interview schedule. Before starting the interviews, we reiterated the research objectives and obtained Behvarzan’s consent for participation. Due to the unavailability of the Behvarzan, the interviews were conducted by telephone and recorded.

The interviews began with a general question: “During your employment, have you had or been interested in electronic health education? Please explain.” It was followed by questions probing their perspectives on the requirements for using this educational method. Data collection continued until data saturation was reached. The interviews were conducted in a calm and informal setting, without preconceptions, biases, or judgments about the correctness or incorrectness of the responses.

Immediately after each interview, we transcribed the recorded content verbatim and briefly documented the points and key topics extracted from each interview in a dedicated form, which included the interviewees’ initial details, the date and time of the interview, and other information. The interviews lasted between 25 and 70 min.

At the end of each interview, we obtained participants’ willingness to answer any potential future questions. If ambiguities arose after transcribing the interview texts, the participants were re-contacted, and new data were added to the interview transcripts.

The interview guide was structured as follows:

1. If we were to list your most important daily responsibilities, what items would you mention?
2. How did your educational model change before and after the COVID-19 pandemic?
3. Have you previously delivered health education to your target group electronically, such as via internet, telephone, SMS, radio, television, or other means? Please describe your experience.
4. In your opinion, what are the advantages and disadvantages of electronic methods?
5. Based on your past experiences and changing conditions, to what extent do you feel the need to provide education through electronic methods?
6. How do you currently assess the requirements for implementing and delivering electronic education?
7. If COVID-19 restrictions were to be lifted, how would you design your educational models?

### Data analysis

Data analysis was performed using the inductive Elo and Kyngas approach, in which the data coding began with specific codes, and by merging them, more general and abstract expressions were formed [23]. Using MAXQDA software, meaningful short words and sentences were identified, and open coding was conducted. The open codes were then merged and placed into relevant subcategories based on semantic similarity. After further reorganization, based on the relationships between the subcategories, the main categories were formed.

To ensure the trustworthiness of the data, credibility, confirmability, and transferability were used [24]. Data credibility was achieved through semi-structured interviews, field reporting, and prolonged engagement with the research topic. Participants and the research team reviewed the categories and examined the data and analysis process. The interviews were conducted within a specific and continuous timeframe with a complete focus on the topic to enhance confirmability, and the data analysis and code categorization process were thoroughly reviewed by experienced qualitative content analysis experts. To improve the transferability of the findings, a clear description of the context, sampling method, participant characteristics, data collection, and analysis process was provided, along with an explanation of the barriers and limitations to enable readers to assess the applicability of the findings in other settings [25].

## Results

The main categories and subcategories are presented in Table 1, and sample quotes from the participants, along with the coding and abstraction of the categories and subcategories, are shown in Table 2.

**Table 1:** Themes and Subthemes”

Themes	Subthemes
1. Technology	o Technical Infrastructure o Content Production o Method of Content Delivery o Content Delivery Channel
2. Facilities and Equipment	o Communication Equipment o Electronic Content Repositories
3. Stakeholders	o Trining of providers o Motivation of providers o Convincing Learners o Target and Reference Groups

According to the participants, clients’ reluctance to physically visit the HHs, especially during the COVID-19 pandemic, has strengthened using e-learning, as the health personnel could utilize virtual education opportunities and provide training to a larger number of individuals in a shorter period.

To present the findings more accurately, they are organized into three main themes and subthemes and relevant explanations.

### Technology

One of the essential requirements for implementing any project is the necessary technology, which also constitutes a significant portion of the requirements for e-learning in this study. This category can be discussed in four subcategories:

- Technical Infrastructure:

Unstable internet connectivity, poor cellular coverage, and lack of a dedicated telephone line for e-learning were the most important issues mentioned as barriers to implementing virtual education or e-learning.

*In our area, when the power goes out, the telephone is cut off first, and then the mobile signal and people's access is limited." (Participant 11)*

b) Content Production:

Entering the realm of virtual education requires the production of relevant and appropriate content. One critical requirement for implementing e-learning programs in HHs is access to reliable multimedia content, which necessitates access to content creation programs and the required equipment. Some active participants in virtual education emphasized the importance of using various audio-visual media for content delivery. Some participants highlighted using short messages accompanied by pictures and short videos as an influential approach.

*"I used various types of pictures, texts, and videos and audio. I think we need to see which model is preferred the most. The type of education should be diverse." (Participant 1)*

*"Video that explains is very effective. They can play the video and do their other tasks as well." (Participant 7)*

c) Content Delivery Methods:

The need for conciseness and gradual presentation of the content was one requirement for the desired content. The value of micro-learning in this situation is evident.

*" There are a series of things that are wrong in the virtual space, where we forward a large number of messages, and the client doesn't have time to read all of them. We need to set goals." (Participant 2)*

*"For virtual education, we shouldn't have too much content - it should be short and catchy. I'll post the next part of the content a few days later - if it's too much, people get overwhelmed." (Participant 7)*

d) Content Delivery Channels:

The main content delivery channels during the pandemic were messaging apps and social media platforms. Considering the capabilities of the available platforms, these are the most suitable and accessible communication channels between healthcare providers and learners. However, using these channels requires technological infrastructure and necessary resources. In addition, using social networks is not possible for all age groups and vulnerable rural groups, including the elderly and the disabled. They cannot receive information in this way. However, social networks and instant messenger software are facing significant success.

*“Most of my neighbors have my phone number*,* and I post a lot of the training*,* and they see it. I also created a WhatsApp group for students*,* where I post the content*,* and there is a chat option there as well.” (Participant 11)*

*”The village council has an Instagram page*,* and we send them posts and stories.” (Participant 13)*

In addition to social media, in some cases, follow-ups were also done via telephone and the SIB (Integrated Health System) platform, particularly about the elderly and foreign nationals, as these groups have more limited smartphone access.

However, an important point to consider as a principle in e-learning is learners’ ability to interact with the instructor and engage in face-to-face sessions, which is not always possible in some of these alternative channels.

### Facilities and equipment

When discussing the requirements for e-learning, the availability of necessary equipment for the educator and the health education learners is essential. Without these facilities, implementing e-learning will not be feasible or face significant challenges. This main category includes two subcategories: communication equipment and electronic content repositories.

- Communication Equipment:

The availability of smartphones is necessary for the educator and the learner. In this study, Behvarzan mentioned that some learners do not have mobile phones or share a phone. This concern is serious for foreign nationals and the elderly. Besides, the personnel also need dedicated equipment, such as smartphones, and using personal mobile phones is not feasible. This issue also applies to telephone lines and some participants expressed reluctance to use their numbers for interaction.

*“Our phones are personal devices*,* and we also buy our internet*,* and this is one of the problems.” (Participant 12)*

*”Many of the elderly and Afghans don’t have phones*,* and we cannot educate online.” (Participant 10)*

The availability of large monitors and multimedia content in the form of dedicated rural educational media or specific programs on provincial networks was recommended by some participants as alternatives to smartphones for learners who cannot use the usual method.

*“The health center should create a rural television that everyone in the village can constantly watch and use.” (Participant 4)*

b) Electronic Content Repositories:

In some cases, participants suggested that content production banks be created, and all Behvarzan could use the standardized content produced.

*“It would be better if the Ministry of Health and Medical Education( MOHME) creates a unified channel that Behvarzan can access*,* and we can access and use the content related to different occasions in a comprehensive and integrated manner.” (Participant 4)*

### Stakeholders

The most important factor for any project is the human. In cases where the necessary facilities and technology exist, but the relevant human resources are not present, implementing the plan will not be feasible. In this study, the importance of human resources was referred to as a requirement for e-learning. This main category also includes four subcategories:

- Training of Providers:

Several issues related to the human resources in HHs are essential in implementing virtual education. The most significant barrier is the lack of personnel’s familiarity with content production methods and related programs. The participants mentioned that specialized individuals should be assigned to content production.

*“Health education is a critical issue because our workload is high*,* so there should be a dedicated person for education who trains a specific number of people and gets feedback from them.” (Participant 9)*

b) Motivating Providers:

The lack of time during the Behvarzan’s presence in the HH meant they had to spend time outside of office hours at home using their equipment for content production or content transfer, and they cited this as an obstacle to the expansion of virtual education. Appropriate incentives should be created to address this. According to the participants, organizational support for pioneers in virtual education is crucial in promoting and assessing the feasibility of e-learning. This support can be provided in overtime pay, employing additional specialized staff at the center, or other financial and non-financial incentives.

*“Using virtual space*,* Behvarzan needs to be supported and encouraged. Motivation should be created so that their activities are effective. Fairness should be observed among all Behvarzan. Those who work and those who pretend to work should be identified.” (Participant 1)*

c) Persuading Learners and Target Groups:

For effective implementation of e-learning, the acceptance of this educational method by the learners is also critical. The cooperation and willingness of the program’s target audience was one of the participants’ concerns as a key part of the educational process. Cultural barriers, especially among foreign national learners, and the unwillingness to provide a mobile phone number for membership in educational groups were among these issues. The participants believed that advertising for e-learning could create more cooperation from the public.

One requirement for implementing e-learning was the necessity of needs assessment from the learners: recognizing their preferences for the type and format of education. The participants believed this survey would help them choose more appropriate content and methods. Additionally, the results would help evaluate the program and its future development.

*” To change the lifestyle*,* we should do an initial assessment*,* start the training based on current conditions*,* and then evaluate it after a year and plan to continue the work.” (Participant 2)*

d) Reference Groups:

Another notable point was that the low literacy level and specific age groups, such as the elderly, make the universal use of electronic content difficult. The emphasis on age groups was not limited to the elderly, and the participants stressed the importance of tailoring the content to the target age group. Besides, attention to specific groups as reference and liaison groups was suggested. For example, mothers, as they are responsible for managing family health, could be utilized as liaisons for virtual education, and this could help address many of the financial problems in providing equipment.

*“Mothers are a critical group and should be considered more for education.” (Participant 12)*

*”As the content manager*,* I created the content categorized by age so it would be more effective. For example*,* for children*,* it’s better to use animation.” (Participant 4)*

*”The principle of education should start from the basics. For example*,* we should teach tooth brushing in schools so that health education is gradually formed. If education starts from the basics and training*,* the person will continue to do it in adulthood and have a healthy lifestyle.” (Participant 2)*

##  Discussion

Our findings with the Behvarzan were categorized into three main categories and ten subcategories, including (1) Technology (technical infrastructure, content production, content delivery methods, and content delivery channels). (2) Facilities and Equipment (communication equipment and electronic content repositories) 3. Stakeholders (training of providers, motivating providers, persuading learners and target groups, and reference groups).

The results reported in the first and second main categories are similar to the findings of the study by Kazemi et al. [26] ,which ranked the influential factors in developing e-learning as hardware and software infrastructure, social communications, and collaborative techniques, teaching content and educational materials, learner evaluation methods, educational fields, and finally, student recruitment methods. The supporting factors, content, and educational tools were reported as influential factors in the success of the e-learning and teaching system in the study by Jafari Yaghoobi et al. [27]. The technology factor was also emphasized for the successful implementation of the virtual education system by Hosein Dargahi et al. [28].

Arasti et al. [29] categorized the factors affecting the success of e-entrepreneurship education into three groups: individual factors (including instructor and student characteristics), systemic factors (including the quality of education and content, internet infrastructure quality, virtual education system quality, and university performance and services), and environmental factors (including interactions and evaluation). The content of the first two categories is similar to the main categories of stakeholders and facilities and equipment, but the environmental characteristics were not mentioned by Behvarzan. The studies highlight the importance of technology and infrastructure, including technical infrastructure, content production, content delivery methods, content delivery channels, hardware and software infrastructure, social communications, and collaborative techniques in e-learning and virtual education systems. Facilities and equipment, such as communication equipment and electronic content repositories, are also crucial, with systemic factors like the quality of education and content, internet infrastructure quality, virtual education system quality, and university performance and services playing a role in e-entrepreneurship education. Additionally, stakeholders, including training providers, motivating providers, persuading learners and target groups, and reference groups, are vital, along with individual factors like instructor and student characteristics. Environmental factors such as interactions and evaluation, are also critical in e-entrepreneurship education.

According to Behvarzan, in the technical infrastructure subcategory, unstable internet connectivity, poor cellular coverage, and the lack of a telephone line for virtual education were the critical barriers to implementing e-learning. From the perspective of the faculty members of Urmia University of Medical Sciences, the technical and support factor was also the most significant obstacle to the development of virtual education in the university. Therefore, by increasing internet speed, replacing outdated systems with new ones, and strengthening technological infrastructure, the inhibiting factors for using virtual education can be reduced [30]. Likewise, Gholamreza Shams et al. [31] referred to issues such as weaknesses in network communications, lack of technical support from the IT unit, and weakness in physical and hardware infrastructure as the infrastructure barriers in e-learning. These studies suggest that institutions and policymakers should prioritize investments in strengthening technical infrastructure to overcome the barriers to implementing e-learning and virtual education. This includes upgrading internet speed, replacing outdated systems, and providing adequate technical support from IT units to ensure seamless network communications and reliable physical and hardware infrastructure. By addressing these technical infrastructure challenges, institutions can reduce the inhibiting factors that hinder the adoption of virtual education, ultimately enhancing the quality and accessibility and improving the overall learning experience for students.

One critical requirement for implementing e-learning programs in HHs is access to reliable multimedia content, necessitating access to content creation programs and equipment. Some active participants in e-learning emphasized the importance of using various audio-visual media for content delivery. Using short messages accompanied by pictures and short videos was also highlighted by the participants as an influential approach. Additionally, there were references to the need for content banks so that all Behvarzan could use the available standardized content.

Taghi Panahi et al. [32]. referred to factors such as quality, awareness-raising, credibility, added value, expertise, motivation, entertainment, uniqueness, relevance, realism, timeliness, creativity, feasibility, shareability, and personalization as essential for the successful acceptance of content in the virtual space by users. Nobakht et al. [33] also mentioned content quality and accessibility as criteria for the quality of e-learning courses.

Regarding the content delivery (teaching) methods, the need for conciseness, gradual and appropriate presentation based on the audience’s level of knowledge, and the instructor’s proficiency were emphasized points. Thani et al. also referred to objectives, content, learning activities, teaching strategies, grouping, materials and resources, time, place, and evaluation as effective in the success of e-learning [34]. These studies suggest that developing and implementing effective e-learning programs in HHs requires considering multimedia content creation, delivery, and accessibility. Specifically, institutions should invest in content creation programs and equipment, utilize diverse audio-visual media, and develop content banks with standardized and high-quality materials. Additionally, content should be designed with user needs, incorporating credibility, motivation, entertainment, and personalization to ensure successful acceptance. Furthermore, instructors should employ concise and gradual teaching methods, tailoring content to the audience’s level of knowledge and utilizing effective teaching strategies, materials, and evaluation methods to ensure the success of e-learning programs. By prioritizing these aspects, HHs can create engaging and effective e-learning experiences that meet the needs of their users.

According to Behvarzan, the main content delivery channels during the COVID-19 pandemic were messaging apps and social media platforms, which seem to be the most suitable and accessible communication channels between healthcare providers and learners. However, using these channels requires the technological infrastructure and necessary software and hardware resources.

The lack of the ability for face-to-face interaction between the learner and the instructor and influential question-and-answer sessions was a weakness, and they emphasized its importance. E-learning can lead to a sense of isolation and separation due to the lack of genuine interactions, as seen in most e-learning platforms, and these feelings can reduce the user’s motivation to learn. The study by Wu et al. [35] showed that a chatbot alongside the e-learning platform can be helpful and reduce the sense of isolation in users.

The availability of necessary facilities and equipment for learners and providers of e-learning is essential. Otherwise, its implementation will not be feasible. The existence of smartphones and, subsequently, home computers connected to the internet are among the required equipment. A study in Uganda showed that tablet-based education is competitive with traditional education in knowledge transfer and leads to learner satisfaction [20].

These studies show that e-learning programs in HHs should leverage popular messaging apps and social media platforms as primary content delivery channels, but ensure that necessary technological infrastructure and resources are in place to support these channels. To mitigate the limitations of e-learning, such as the lack of face-to-face interaction and a sense of isolation, HHs can consider incorporating innovative solutions like chatbots to facilitate user engagement and motivation. Furthermore, the availability of essential facilities and equipment, including smartphones and internet-connected devices, is crucial for the successful implementation of e-learning programs. By acknowledging these factors, HHs can develop effective e-learning strategies that promote learner satisfaction and knowledge transfer, ultimately enhancing the quality of healthcare education.

For implementing any project, the critical factor is human resources. In e-learning, attention to human resources is also of great importance, and one of the most significant barriers is the lack of familiarity of the personnel with content production methods and related programs. The participants emphasized the production of content by specialists. Therefore, to strengthen the knowledge and skills of Behvarzan in e-learning, these individuals should receive continuous e-learning training. Robert Bollinger et al. also recommended providing specialized training for instructors and students to increase their skills in using e-learning platforms [36].

According to the participants, organizational support for active individuals in e-learning is critical in promoting and strengthening e-learning. Karimian and Farrokhi introduced the steps for developing virtual education in medical universities, identified training of human resources, setting regulations, and encouraging and motivating them as the main strategies in developing virtual education [37].

These studies emphasize that the success of e-learning programs in HHs hinges on the development and empowerment of human resources, particularly in content production and e-learning platform proficiency. To overcome the barriers of inadequate familiarity with e-learning methods, HHs should invest in continuous training and capacity-building programs for Behvarzan, enabling them to produce high-quality content and effectively utilize e-learning platforms.

Behvarzan also emphasized the need to persuade learners and target groups. The cooperation and willingness of the program’s target audience were participant’s concerns as one of the main components of the educational process. One requirement for implementing e-learning was the needs assessment from the learners to identify educational topics and design them accordingly. The participants believed that this survey would help them choose more appropriate content and methods, and in the next step, they could use the evaluation results to improve and strengthen the program. In another study, individual characteristics of the learner, cultural considerations, the relationship between learners, the relationship between the learner and the teacher, the learner’s prior knowledge, and the learner’s interaction with the environment were influential components of the quality of e-learning [38].

Behvarzan also emphasized tailoring the content to the target age group. Besides, attention to specific groups as reference and liaison groups was suggested. For example, mothers could be utilized as liaisons for e-learning, which would enhance the effectiveness of the training, and also utilize teenagers as health ambassadors to strengthen family engagement and facilitate the rapid transmission of health information. In this regard, Mahdavi-Nasab et al. emphasized the analysis of the learner as one of the influential components of the effectiveness of e-learning [39]. These studies suggest that the success of e-learning programs in HHs relies on understanding and catering to the needs and characteristics of the target audience, including learners and their families. HHs should conduct needs assessments to identify educational topics and design content accordingly, considering individual characteristics, cultural considerations, prior knowledge, and environmental interactions. Moreover, tailoring content to specific age groups and utilizing reference and liaison groups, such as mothers and teenagers, can enhance the effectiveness of e-learning and facilitate the transmission of health information. By prioritizing learner analysis and engagement, HHs can create e-learning programs that are responsive to the needs of their target audience, ultimately leading to improved health outcomes and more effective health education.

Among the limitations of this study, one can refer to the unwillingness of some Behvarzan to be interviewed due to fear of their names being disclosed, addressed by assuring them of the confidentiality of the participants’ names in the initial contact. Additionally, since this study was conducted in Karaj City, there is a presumption that the people in this region and Behvarzan have access to more resources, and the results may not accurately reflect the deprived provinces. The study’s strength was the participants’ distribution in areas with diverse geographical characteristics.

## Policy Brief

1. The Ministry of Communications and Information Technology should establish the infrastructure for improved mobile network coverage and high-speed internet in rural areas.
2. The Health Deputy of the MoHME should incorporate electronic education into Behvarzes’ job description and allocate financial incentives accordingly.
3. Medical Universities should provide each HH with a smartphone and an organizational telephone line.
4. A fast, user-friendly, and popular online communication platform should be introduced to Behvarzes and rural populations.
5. The MoHME should introduce an offline communication platform (such as group SMS) to Behvarzes, with necessary coordination facilitated by district health networks.
6. Behvarz Training Centers within health networks should incorporate electronic education-related courses into the Behvarz training curriculum.
7. The Information Technology unit within the health network should train Behvarzes on delivering electronic education through the designated online communication platform(s).
8. A working group for producing multimedia content (tailored to age groups and diseases) should be established within the MoHME and made available to health networks.
9. Provincial and district content production working groups should focus solely on issues specific to their geographical area to avoid duplication of efforts.
10. Electronic education for elderly target groups and foreign nationals (who lack access to or knowledge of relevant equipment) should be conducted via telephone calls by Behvarzes.
11. Behvarzes should collaborate with health ambassadors (an interested member from each family) as liaisons for electronic education and disseminate information.
12. Medical Universities should coordinate with provincial broadcasting services to provide health education on specific days and times of the week and during special occasions (to cover individuals beyond the reach of Behvarzes).

These recommendations encompass a comprehensive approach to implementing electronic health education in rural areas, addressing infrastructure, policy, training, content development, and outreach strategies. They emphasize the need for multi-sectoral collaboration and adapting existing health systems to incorporate digital technologies effectively.

##  Conclusion

Behvarzes perspectives provide valuable guidance for policymakers, program planners, and healthcare organizations seeking to transition health education to electronic/digital platforms. To operationalize the delivery of electronic health education by Behvarzes to the public, we recommend communication and information infrastructure related to electronic education be established, and necessary equipment and facilities be provided to stakeholders. Additionally, it is essential to create a working group at the MoHME level, comprising experts in media literacy, communications, information technology, and health education. This group should design precise processes for electronic health education (both online and offline) from the MoHME to HHs and create and introduce standardized and appropriate content. Simultaneously, necessary training and incentives should be provided for Behvarzes and target groups regarding electronic education, and communication channels should be established on suitable platforms to facilitate mutual interaction between Behvarzes and the public, enabling the transfer of targeted educational content.

## Data Availability

The datasets used and/or analyzed during the current study available from the ‎corresponding ‎author ‎on reasonable request. The entire dataset is in Farsi language. The ‎Data can be available in ‎English ‎language for the readers and make available from the ‎corresponding author on reasonable ‎request.‎.
